# Comparison of Antimicrobial Susceptibility Profiles of Thermotolerant *Campylobacter* spp. Isolated from Human and Poultry Samples in Georgia (Caucasus)

**DOI:** 10.3390/antibiotics11101419

**Published:** 2022-10-17

**Authors:** Maia Metreveli, Salome Bulia, Liana Tevzadze, Shota Tsanava, Michael Zarske, Juan Cruz Goenaga, Sandra Preuß, Giorgi Lomidze, Stylianos Koulouris, Paata Imnadze, Kerstin Stingl

**Affiliations:** 1 Faculty of Medicine, Ivane Javakhishvili Tbilisi State University, 0179 Tbilisi, Georgia; 2 Department of Gastroenteric Infection Diseases, Tbilisi Children Infectious Diseases Clinical Hospital, 0171 Tbilisi, Georgia; 3 National Center for Disease Control and Public Health, 0198 Tbilisi, Georgia; 4 National Reference Laboratory for *Campylobacter*, Department of Biological Safety, German Federal Institute for Risk Assessment, 12277 Berlin, Germany; 5 Faculty of Medicine, European University, 0189 Tbilisi, Georgia; 6 European Commission, Directorate General for Health and Food Safety (DG-SANTE), 1049 Brussels, Belgium

**Keywords:** EUCAMP3, microdilution, cgMLST, backyard chicken, whole-genome sequencing, resistance determinant, campylobacteriosis, gastroenteritis, WGS

## Abstract

Antimicrobial resistance remains a public health concern globally. This study presents antimicrobial resistance by microdilution and genetic diversity by the whole-genome sequencing of *Campylobacter* spp. from human and poultry samples isolated in Georgia in 2020/2021. The major species in poultry samples was *C. coli*, while *C. jejuni* was preferentially isolated from human samples. Resistance against tetracycline was highest (100%) in *C. coli* from industrial chicken and lowest in *C. jejuni* from clinical isolates (36%), while resistance against ciprofloxacin varied from 80% in *C. jejuni* from backyard chicken to 100% in *C. jejuni* and *C. coli* from industrial chicken. The point mutations in *gyrA* (T86I) and *tet* (O) genes were detected as resistance determinants for (fluoro-)quinolone or tetracycline resistance, respectively. Ertapenem resistance is still enigmatic. All isolates displayed sensitivity towards erythromycin, gentamicin and chloramphenicol. Multi-resistance was more frequently observed in *C. coli* than in *C. jejuni*, irrespective of the isolation matrix, and in chicken isolates compared to human isolates, independent of the *Campylobacter* species. The Georgian strains showed high variability of multi-locus sequence types (ST), including novel STs. This study provides the first antibiotic resistance data from *Campylobacter* spp. in Georgia and addresses the need for follow-up monitoring programs.

## 1. Introduction

The emergence and spread of multi-resistant bacteria continues to be a global public health concern. In the European Economic Area (EEA), it was estimated that more than 670,000 diseases were caused by antimicrobial-resistant (AMR) bacteria yearly, with about 33,000 associated deaths [1].

Campylobacteriosis is a disease caused by thermotolerant *Campylobacter* spp. It is one of the four major causes of diarrhea worldwide, and is considered to be the most common cause of bacterial food-borne human gastroenteritis [2]. *Campylobacter* species are motile, curved, microaerobic, Gram-negative rods that commonly reside in the intestinal tract of many wild and domestic warm-blooded animals.

Although campylobacteriosis is mostly self-limiting, recent reports showed that a substantial proportion (31%) of reported *Campylobacter* infections have been treated with antibiotics [3], probably those infections with severe outcome. Concordantly, a considerable number of 21% of the reported campylobacteriosis cases resulted in hospitalization in the EU in 2020, while for comparison, salmonellosis led to 29.9% and infections by shiga-toxin-producing *E. coli* to 40.9% of hospitalization [4].

Based on the joint report of European Food Safety Authority (EFSA), European Centre for Disease Prevention and Control (ECDC), European Medicines Agency (EMA) and Organization for Economic Co-operation and Development (OECD) the overall consumption of antibiotics in humans decreased by 23% and in food-producing animals by 43% between 2011 and 2020 in the EEA [5]. Harmonized AMR key indicator bacteria, such as fully susceptible *Escherichia coli* for food-producing animals and Methicillin-resistant *Staphylococcus aureus* (MRSA) for humans varied depending on the country and the years. In the majority of the countries, the proportion of fully susceptible *E. coli* increased and MRSA decreased between 2014 and 2018, being in-line with reduced use of antibiotics [6]. However, the percentage of *E. coli* from human samples resistant against third-generation cephalosporins increased in half of the countries and decreased in the other half. Of particular concern is the increase in carbapenem resistance with, e.g., almost a quarter of EU/EEA countries reporting at least 10% carbapenem-resistant *K. pneumoniae* [1]. Carbapenems are not authorized for use in veterinary medicine in the EU [7] and in Georgia [8]. Combined resistance to both ciprofloxacin and erythromycin, which is considered critically important for treatment of campylobacteriosis, was marginal with 0.5% in *C. jejuni* and still low with 8.9% in *C. coli* in 2020. However, relatively high levels of combined resistance were reported by Finland and Portugal for *C. coli* (36.8–40.6%) [6]. In a global world, emerging resistant strains identified at one location can be spread around the world, thus, the issue requires a global systematic approach and international action [9].

AMR surveillance data from Georgia are scarce in the public health system and absent at the food production and veterinary sectors. The Central Asian and European Surveillance of Antimicrobial Resistance (CAESAR) 2019 report [10] described resistance data gathered in twelve countries of the WHO European Region including Georgia. Data from Georgia were assessed reliable with limitations of small number of samples, focus on samples from the capital and lack of harmonized AST guidelines [10]. Data on *Campylobacter* spp. were lacking.

Antimicrobial resistance monitoring in *Campylobacter* from poultry samples in Europe is performed based on the regulation 2003/99/EC, laying down the monitoring of zoonoses and zoonotic agents isolated from distinct food and animal matrices and their characterization using harmonized panels of antimicrobial substances [11]. In several countries, an increase in resistance in *C. jejuni* from broilers against tetracycline and ciprofloxacin was detected. In addition, *C. jejuni* isolates from human samples also showed increasing resistance to these antimicrobials [6].

On the way of EU integration, the regulation for monitoring of zoonoses and zoonotic agents based on the 2003/99/EC went into force in Georgia in 2020. According to this regulation monitoring of antimicrobial resistance has to be carried out at primary production level and/or at other stages of the food chain. The regulation covers zoonoses including *Campylobacter* spp.; however, implementation of the regulation is not in action yet.

Our study presents first data on genetic diversity of *Campylobacter* spp. strains from human stool and poultry samples isolated in Georgia based on whole genome sequencing analysis and identifies antimicrobial resistance patterns of *C. jejuni* and *C. coli* including their genetic determinants. The study encourages future monitoring programs for in-depth analysis of thermotolerant *Campylobacter* spp. in Georgia in order to improve food safety.

## 2. Materials and Methods

### 2.1. Sampling and Transport

In total, 160 *Campylobacter* isolates were obtained from chicken cecal samples from February 2020 until September 2021 in Georgia. The 110 so-called “backyard” chicken samples were gathered at the Digomi live animal market in Tbilisi, where poultry is sold reared at small farms and households from all over the country and directly processed on the market slaughterhouse. Another 50 *Campylobacter* strains were isolated from samples collected at a medium-sized ‘intensive-rear’ poultry farm slaughterhouse, located at the eastern part of Georgia. In addition, 382 human stool samples had been previously collected from July 2020 to July 2021 at the Tbilisi Children Infectious Diseases Clinical Hospital from hospitalized children with diarrhea, from which 60 were positive for *Campylobacter* spp. [12]. Human stool samples were transported on Cary-Blair medium (Biolife Italiana srl, Milan, Italy) at cooling temperatures without microaerobic conditions and analyzed within 24 h. Chicken cecal samples were transported in plastic bags on ice and analyzed within 3–6 h after sampling.

### 2.2. Detection and Phenotypic Identification of Campylobacter spp.

*Campylobacter* detection was performed according to ISO 10272-1:2017 part C on modified charcoal cefoperazone deoxycholate agar (mCCDA) (Thermo Fisher Specialty Diagnostics Ltd., Hampshire, UK). For the clinical samples, *Campylobacter* Chromogenic agar *Campylobacter* (CHROMagar, France) was applied as an additional second selective medium to increase sensitivity [12]. Less than 20% of the clinical samples were also enriched with Preston broth (Biolife Italiana S.r.l., Milan, Italy) [13], but the results showed no enhanced detection [12].

Ceca were aseptically cut and the content mixed. One 1 µL loop of the cecal material was directly streaked on the mCCDA agar plate and distributed over the surface by using a fresh loop. The human stool samples were treated similarly but in addition to mCCDA a second selective plate was used in parallel. Incubation was performed at 42 °C in a microaerobic gas mixture consisting of 85% nitrogen, 10% carbon dioxide and 5% oxygen (LTD Argoni, Tbilisi, Georgia).

Suspicious colonies were sub-cultured on Columbia Blood Agar (ColbA; AES Laboratories, Bruz Cedex, France). Confirmation of colonies was initially performed applying the Biomerieux system ApiCampy (Biomerieux Inc, Marcy-l’Etoile, Lyon, France), consisting of 20 microtubes containing dehydrated substances. One half contained enzymatic tests and the other half substrates for assimilation or inhibition. In the latter, growth of bacteria is monitored. The specific pattern of growth and presence of enzymatic activity is used as read-outs for identification of bacteria. In addition, colonies were observed by microscopy after Gram-straining. All isolates were stored at −80 °C for further characterization.

### 2.3. Confirmation of Campylobacter Species and Differentiation by Real-Time PCR Analysis

At the National Reference Laboratory for *Campylobacter* at BfR the 220 strains, from which 160 were derived from chicken and 60 from human sources, were re-cultured on ColbA for 48 h under microaerobic atmosphere. In case no growth or some contamination was obtained, a parallel enrichment in Bolton broth (Oxoid, Thermo Fisher Scientific Inc., Waltham, MA, USA) with 5% lysed defibrillated horse blood (Oxoid, Thermo Fisher Scientific Inc., Waltham, MA, USA) was streaked on mCCDA and incubated for another 48 h. Single suspected colonies were sub-cultured on ColbA and incubated 24 h under similar conditions.

Isolates of *Campylobacter* spp. were species-differentiated by real-time PCR [14]. For this purpose, cell material of isolates was resuspended in 5% Chelex 100 resin (Bio-Rad Laboratories GmbH, Feldkirchen, Germany) and heated for 15 min at 95 °C for thermal lysis. Cell debris was centrifuged for 5 min at 14,000× *g*, and the supernatant containing bacterial DNA was used for PCR analysis at a volume of 2.5 µL after 1:100 dilution. Oligos and dark-quenched (DQ) probes in HPLC-grade were as follows: for *C. jejuni*, *mapA*-F, 5′-CTG GTG GTT TTG AAG CAA AGA TT-3′, *mapA*-R, 5′-CAA TAC CAG TGT CTA AAG TGC GTT TAT-3′ and *mapA*-probe, 5′FAM-TTG AAT TCC AAC ATC GCT AAT GTA TAA AAG CCC TTT-3′DQ; for *C. coli*, *ceuE*-F, 5′-AAG CTC TTA TTG TTC TAA CCA ATT CTA ACA-3′, *ceuE*-R, 5′-TCA TCC ACA GCA TTG ATT CCT AA-3′ and *ceuE*-probe, 5′JOE-TTG GAC CTC AAT CTC GCT TTG GAA TCA TT-DQ; for *C. lari*, *gyrA*1-F1, 5′-GAT AAA GAT ACG GTT GAT TTT GTA CC-3′, *gyrA*1-R1, 5′-CAG CTA TAC CAC TTG ATC CAT TAA G-3′, *gyrA*1-F2, 5′-GAT AAA GAT ACA GTT GAT TTT ATA CC-3′, *gyrA*1-R2, 5′-TGC AAT ACC ACT TGA ACC ATT A-3′ and *gyrA*1-probe, 5′Cy5-TTA TGA TGA TTC TAT GAG TGA GCC TGA TG-DQ; for the internal amplification control, IPC-ntb2-F, 5′-ACC ACA ATG CCA GAG TGA CAA C-3′, IPC-ntb2-R, 5′-TAC CTG GTC TCC AGC TTT CAG TT-3′ and IPC-ntb2-probe, 5′TAMRA-CAC GCG CAT GAA GTT AGG GGA CCA-DQ. Note that *gyrA*1-F2 bears one base exchange T3A relative to the original publication due to oligo optimization for the validation study [15]. Oligos at final concentrations of 300 nM (Sigma Aldrich, Steinheim, Germany), 100 nM dark-quenched probes (TIB MOLBIOL, Berlin, Germany) and 1 U of Platinum Taq DNA polymerase (Thermo Fisher Scientific Inc., Waltham, MA, USA) were used. As amplification control, 25 copies of the IPC-ntb2 plasmid [16] was added per PCR reaction.

### 2.4. Antimicrobial Susceptibility Testing

Isolates were tested for AMR according to the prescriptions given in Commission Implementing Decision (CID) (EU) 2020/1729 (European Commission, 2020) [17]. Broth microdilution susceptibility testing was performed according to M45-A (Clinical and Laboratory Standards Institute [CLSI], 2015) [18] and VET06 (CLSI, 2017) [19] with the in-house validated modification of the use of fetal calf serum (PAN-Biotech GmbH, Aidenbach, Germany) instead of lysed horse blood in the culture medium for improved readability of *Campylobacter* growth. For this purpose, strains were subcultured on Columbia blood agar for 24 ± 2 h at 42 °C under microaerobic atmosphere (5% O_2_, 10% CO_2_, 85% N_2_). Cation-supplemented Mueller–Hinton broth (TREK Diagnostic Systems, United Kingdom) supplemented with 5% fetal calf serum was inoculated with 2–8 × 10^5^ colony forming units/mL. Minimum inhibitory concentrations (MICs) were determined using the European standardized microtiter plate format EUCAMP3 (TREK Diagnostic Systems). Antimicrobials tested included chloramphenicol (CHL; 2–64 mg/L), erythromycin (ERY; 1–512 mg/L), gentamicin (GEN; 0.25–16 mg/L), ciprofloxacin (CIP; 0.12–32 mg/L), tetracycline (TET; 0.5–64 mg/L) and ertapenem (ETP; 0.12–4 mg/L). Epidemiological cut-off values (ECOFFs) were taken from the European Committee for Antimicrobial Susceptibility Testing (EUCAST; https://mic.eucast.org/Eucast2 (accessed on 7 September 2022)) laid down in the CID 2020/1729. For *C.* spp. ECOFFs were as follows: 16 mg/L (CHL), 0.5 mg/L (CIP), 0.5 mg/L (ETP) and 2 mg/L (GEN). For ERY and TET, species-specific cut-off values were used (4 or 8 mg/L (ERY) and 1 or 2 mg/L (TET) for *C. jejuni* or *C. coli*, respectively). Incubation was performed for 44 ± 4 h at 37 °C under microaerobic atmosphere. MICs (mg/L) were semi-automatically analyzed using the Sensititre Vizion system (TREK Diagnostic Systems), which has an integrated camera and a mirror, recording a translucent picture from the microtiter plates. The MIC data were stored and exported using Sensi Vizion Software 2.0 (MCS Diagnostics BV, Swalmen, The Netherlands).

### 2.5. NGS Methodology

Genomic DNA was extracted from *Campylobacter* strains sub-cultured overnight using the PureLink Genomic DNA Mini Kit (Thermo Fisher Scientific, Waltham MA, USA) according to the manufacturer’s instructions. DNA was fluorimetrically quantified by Qubit 3.0 Fluorometer (dsDNA HS Assay Kit 0.2–100 ng; Thermo Fisher Scientific, Waltham, MA, USA). The quality of the DNA was evaluated by spectral analysis (NanoDrop Spectrophotometer, Thermo Fisher Scientific, Waltham, MA, USA). DNA libraries were prepared using the Illumina DNA Prep, (M) Tagmentation Kit according to manufacturer’s instructions (Illumina Inc., San Diego, CA, USA) but with using half of the volume of all reagents. Paired-end sequencing was performed on the Illumina MiSeq System (2 × 151 cycles) using the MiSeq Reagent Kit v3 (600 cycles, Illumina Inc., San Diego, CA, USA). Trimming and de novo assembly of raw reads were carried out using the AQUAMIS pipeline v1.3.8 (https://gitlab.com/bfr_bioinformatics/AQUAMIS (accessed on 7 September 2022)). The quality of the assembled genome contigs was automatically evaluated using the teQuilR in-house pipeline. Sequences were published within the BioProject No. PRJNA844526 at the NCBI sequence read archive (SRA). Ridom Seqsphere+ v8.2.0 (Ridom, Muenster, Germany) was used to perform phylogenetic analysis on assembled genome contigs using the cgMLST scheme of 1343 gene targets previously defined [20] with 98% required identity and 98% required percentage of coverage to one of the alleles of the reference sequence NC_002163.1.gb (*C. jejuni* NCTC 11168). At least 95% “good targets” were found for cgMLST-based analysis using the previously proposed cgMLST scheme. New MLST alleles and MLST-ST types were uploaded to PubMLST (www.pubmlst.org). Prediction of antimicrobial resistance determinants and plasmid markers within assembled genome contigs was performed by using the BakCharak pipeline v2.0 (https://gitlab.com/bfr_bioinformatics/bakcharak (accessed on 7 September 2022)). Tools in the pipeline include ABRicate v1.0.1 (https://github.com/tseemann/abricate (accessed on 7 September 2022)) and AMRFinderPlus v3.6.15 [21] and its associated database for antimicrobial resistance determinant, as well as Platon v1.1.0 for plasmid prediction (https://github.com/oschwengers/platon (accessed on 7 September 2022), [22] and plasmid blaster, a tool that performs a BLAST analysis against the NCBI RefSeq plasmid database. BLAST results were filtered with at least 20% coverage of the contig length.

### 2.6. Statistical Analyses

Isolates were categorized into susceptible and resistant, using the epidemiological cut-off values as mentioned in Section 2.4. The dependent variable was resistant vs. susceptible (reference category) to the antimicrobial in question. In addition to the individual antimicrobial, an outcome variable “2-3-fold resistance” was defined for an isolate resistant against two or three tested antimicrobials. This means that first, isolates were categorized according to their MIC and the epidemiological cut-off value (ECOFF) as sensitive or resistant towards every individual antimicrobial. Second, the number of resistances per isolate was counted and those with 2 or more resistances were defined as displaying “2-3-fold resistance”.

Multiple logistic regression with forward selection was used to establish independent predictors for tetracycline resistance (variables of matrix source (human vs. chicken (reference category)) and bacterial species (*C. coli* vs. *C. jejuni* (reference category)) were included). A Nagelkerke R Square and a non-standardized beta coefficient (B) were calculated. An odds ratio with 95% confidence interval (CI) was calculated as an exponential of the B coefficient (Exp [B]).

For all analyses, *p*-values of less than 0.05 were considered statistically significant. Statistical analyses were performed using SPSS (IBM SPSS Statistics for Windows, Version 21.0. Armonk, NY, USA: IBM Corp).

## 3. Results

### 3.1. Collection of Campylobacter spp. Strains and Identification of Species

*Campylobacter* spp. isolates from chicken cecal content were obtained from February 2020 until September 2021. “Backyard” chicken samples aged between several days to one year were collected from chicken reared on small farms and in households all over the country and sold at a live market in Tbilisi. In addition, *Campylobacter* strains were isolated from samples collected at a medium-sized industrial poultry slaughterhouse, located at the eastern part of Georgia and supplying Tbilisi with fresh chicken meat. Those chickens were “standardized” with an age between 38 and 42 days. In addition, human stool isolates had been previously collected from hospitalized children with diarrhea from July 2020 to July 2021 [12]. Hence, the samples correlated in time and space. From a total of 220 isolates—160 derived from chicken and 60 from human sources (Supplementary Materials Table S1)—sixteen were non-culturable after transport to BfR. However, from these sixteen non-culturable samples, *Campylobacter* spp. were still detectable by real-time PCR in twelve of the enrichment inoculums, showing either *C. coli* (4/12) or *C. jejuni* (3/12) in seven cases and mixed cultures of *C. coli* and *C. jejuni* in five cases (41%, *n* = 5/12).

**Figure 1:**
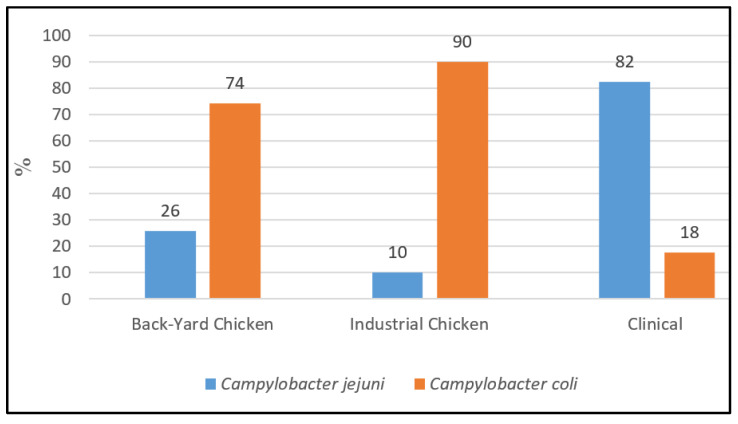
*Campylobacter* species distribution (%) in poultry and human samples.

Out of 204 strains re-cultured, 37.7% (*n* = 77) were identified as *C. jejuni* and 62.3% (*n* = 127) as *C. coli* applying real-time PCR [14]. The distribution of isolated species differed between human stool samples and cecal chicken samples (Figure 1).

From the isolates of backyard chicken, 25.8% were identified as *C. jejuni* (*n* = 25/97) and 74.2% (*n* = 72/97) as *C. coli*; in cecal samples from industrial chicken, *C. coli* was even more dominant with 90% (*n* = 45/50). In contrast, out of 57 clinical strains of children stool samples, 82.5% (*n* = 47/57) were identified as *C. jejuni* and 17.5% (*n* = 10/57) as *C. coli* (Figure 1) [12].

### 3.2. Prevalence of Antimicrobial Resistance in Campylobacter Isolates

All isolates were tested for their resistance to the six antimicrobials chloramphenicol, ciprofloxacin, ertapenem, erythromycin, gentamicin and tetracycline according to the European standardized EUCAMP3 plate format. Results from resistance testing are shown in Table 1. All tested strains were sensitive towards gentamicin, erythromycin and chloramphenicol. Resistance in both human and poultry isolates and in both bacterial species was highest against ciprofloxacin and tetracycline.

Both human and poultry *C. coli* strains showed resistance against ertapenem—37% of the strains from backyard chicken, 60% of human isolates and 82% of industrial chicken strains, while *C. jejuni* isolates were fully susceptible to this antimicrobial. Among the ertapenem-resistant *C. coli*, 89% (*n* = 62) had a MIC value of 1 µg/mL, just above the current cut-off value, 10% (*n* = 7) displayed a MIC of 2 µg/mL and a single strain had a MIC of 4 µg/mL. From the strains with MIC values ≥2 µg/mL ETP, three were derived from human samples, four from backyard chicken and one from industrial chicken.

**Figure 2:**
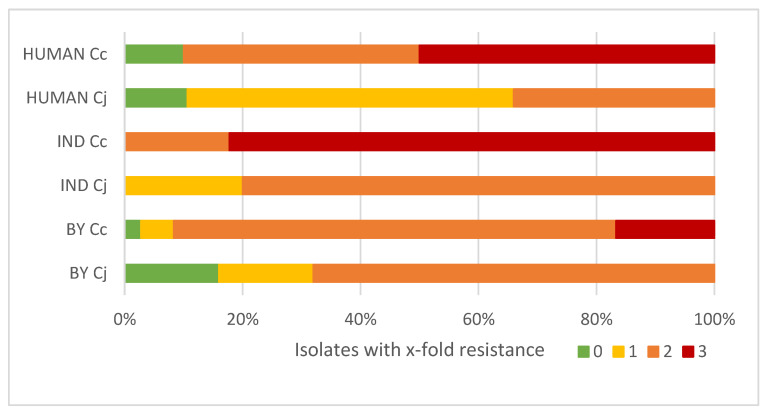
Resistance against antimicrobial classes in *Campylobacter* spp. isolates from different sources. Green, sensitive; yellow, 1-fold-resistant; orange, 2-fold-resistant; red, 3-fold-resistant. *Cj*, *C. jejuni*; *Cc*, *C. coli*; BY, backyard chicken; IND, industrial chicken; HUMAN, human isolates. Resistances against individual antimicrobials detailed in Table 1 were counted per isolate and percentage of isolates with resistances against x-fold antimicrobial classes are depicted here.

Overall, isolates of *C. coli* were less frequently fully susceptible (3/127, 2.4%) than isolates of *C. jejuni* (9/77, 11.6%), with each six strains isolated from backyard poultry and human samples and lack of susceptible strains among the industrial isolates (Figure 2).

*C. coli* were more likely resistant—compared to *C. jejuni*-against ciprofloxacin (OR 5.1, 95% CI 1.6–16.7) and tetracycline (OR 4.6, 95% CI 2.5–8.8). In addition, isolates from clinical samples were less likely resistant to tetracycline compared to chicken isolates (OR 0.18, 95% CI 0.1–0.4). No statistically significant difference was observed for resistance to ciprofloxacin between human and poultry isolates.

Overall, *C. coli* was 18.5 times more likely resistant against two or more antibiotics compared to *C. jejuni* (OR 18.5, 95% CI 7.7–44.8). The same was observed in clinical isolates, where *C. coli* was 17.4 times more likely resistant to two or more antimicrobials than *C. jejuni* (OR 17.4, 95% CI 2.03–150.1); for poultry samples *C. coli* OR showed 7.9 times more probability to have resistance against two or more antibacterial agents compared to *C. jejuni* (OR 7.9,95% CI 2.6–24.6).

There was a significant association of multi-resistance probability with isolation source in *C. jejuni* strains. In particular, the probability of resistance against two or more antimicrobials for chicken isolates of *C. jejuni* was 4.5 times higher compared to human isolates (OR 4.5, 95% CI 1.7–12.1); however, we did not find a significant association between clinical and chicken isolates for *C. coli* species, probably due to low number of *C. coli* isolates from human stool samples. Additionally, no statistically significant difference was found for the presence of two or more resistances in *C. jejuni* or in *C. coli* isolates from industrial compared to backyard chicken.

Variables of bacterial species and isolates were subjected to logistic regression analysis to test association with resistance to two or more antimicrobials as dependent variables. Both variables were retained in the final model as independent variables. The Nagelkerke pseudo R squared was 0.435 indicating that more than 43% of the variability of dependent variables is due to the independent variables model.

Multi-variate logistic regression was performed with two variables which showed significant association with tetracycline resistance. Both variables, bacterial species and sample sources were retained into final model as independent predictors. The regression model can explain more than 20% of the variation in the dependent variable (tetracycline resistance), according to the Nagelkerke pseudo R squared of 0.204. (Table 2). In other words, the predictive model, consisting of the variables “bacterial species” and “sample sources”, can explain 20% of the variability of the dependent variable “tetracycline resistance”. Alternatively, this means, that the remaining 80% of the variability of the dependent variable could be explained with variables, that were not measured within the study and/or are not identified as a possible predictor for the outcome variable. Nagelkerkes R squared 43% for the dependent variable “2-3-fold resistance” can be interpreted in the same way.

### 3.3. Campylobacter spp. Isolates Are Phylogenetically Diverse

We additionally analyzed forty *Campylobacter* strains by whole-genome sequencing, twenty derived from poultry and another twenty from human samples, approximately each ten *C. jejuni* and *C. coli* per matrix. The poultry isolates were both from backyard samples (*n* = 14) and from industrial chicken (*n* = 6). After de novo assembly of the raw reads, multi-locus sequence type analysis (MLST, based on 7 housekeeping genes) and, for more precise resolution, the core-genome MLST (cgMLST) scheme based on the comparison of 1343 gene alleles was used for phylogenetic analysis. Missing cgMLST loci were pairwise ignored.

**Figure 3:**
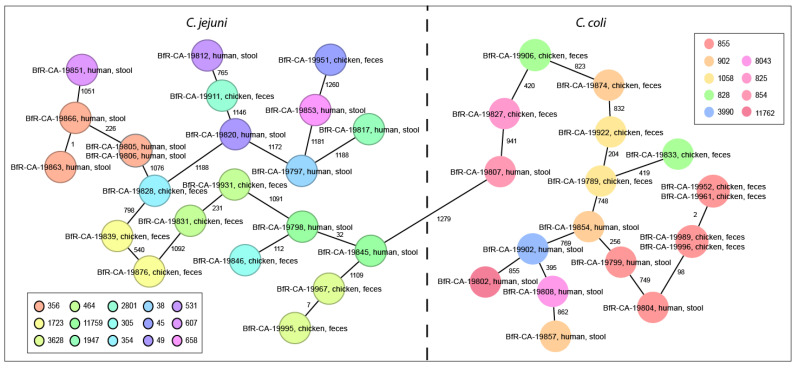
Whole-genome sequences of the isolates from chicken and human samples in Georgia displayed high variability. Minimum spanning tree of cgMLST analysis was based on 1343 core genes defined previously [20]. Missing alleles were pairwise ignored. Each colored circle with (*C. jejuni*) our without frame (*C. coli*) represents an ST-type of the 7 housekeeping genes MLST scheme as depicted in the inlay boxes per species. Numbers next to the connecting lines illustrate the number of allele differences analyzed by cgMLST between nearest neighbors. One new *uncA* allele and two new ST-types were found. More details, including all ST-types are shown in Supplementary Materials Table S2.

As expected, we obtained a high variability of multi-locus sequence types (ST, *n* = 24), including three strains with either unknown *uncA* allele and/or unknown ST-type. The *C. jejuni* (*n* = 22) belonged to 15 different ST-types, while the *C. coli* (*n* = 18) displayed 9 different ST-types (Figure 3). The most frequent ST-types were ST-855 (*n* = 6), ST-356 (*n* = 4), and ST-902 (*n* = 3). The *C. coli* ST-types most frequently grouped within the common clonal complex ST-828 (17/18). Supplementary Materials Table S2 highlights new ST-types and their respective allelic combinations not previously reported in the PubMLST database as well as the metadata of the dataset.

Within the limited number of sequenced strains, we even found three sequence clusters. One of this clusters (ST-855) included four highly similar *C. coli* strains from industrial chicken, collected in June/July 2021 during three independent samplings, with maximal two cgMLST allele differences. Two further *C. jejuni* clusters with each two strains identified among the human isolates belonged both to ST-type 356 and were separated from each other by 226 allele difference. One of these clusters included two *C. jejuni* strains isolated from children in September and October 2021, harboring identical pairwise cgMLST. The other cluster included two *C. jejuni* strains isolated from children in July and September 2021.

Eighteen *Campylobacter* isolates (45%) putatively carried plasmids (Supplementary Materials Table S2), since contigs of the whole genome assembly were predicted as epichromosomal elements by Platon and BLAST analysis using the NCBI RefSeq plasmid database. All plasmids had at least 20% coverage of homology to known *Campylobacter* spp. plasmids (Supplementary Materials Table S3), except for BfR-CA-19911, which harbored a small plasmid without any match in the RefSeq database.

### 3.4. Detection of Antimicrobial Resistance Genes

Whole-genome sequencing analysis also revealed several resistance genes, responsible for the observed phenotypes. The presence of the *tet*(O) gene, which mediates resistance to tetracycline, was detected in all tetracycline-resistant strains (70%, *n* = 28/40). The most common mutation in the *gyrA* gene (T86I) was identified in all ciprofloxacin-resistant isolates (90% (*n* = 36/40)). The presence of *bla*_OXA-61_ family genes (OXA-193, OXA-452, OXA-460, OXA-461, OXA-489, OXA-594), which confer resistance to beta-lactams, was observed in 75% (*n* = 30/37) of strains. In addition, we found the *aadE-Cc* gene in three *C. coli*, putatively conferring streptomycin resistance. Streptomycin and ampicillin are not part of EUCAMP3 plate format, so the phenotype was not confirmed. The AMRFinderPlus database also annotated the mutation 50S_L22_A103V of the L22 ribosomal protein as a putative resistance marker for macrolide resistance in 30% (*n* = 12/37) of the strains; however, all isolates were sensitive towards erythromycin. The resistance mechanism against ertapenem is still unknown. According to Platon prediction, all resistance determinants were chromosomally located.

## 4. Discussion

EU countries have made significant strides in developing and implementing national monitoring plans on antimicrobial resistance [6]; however, in Georgia, monitoring programs are still lacking.

Our study results on antibiotic resistance in Georgian *Campylobacter* spp. isolates from chicken show similarities to the AMR data profiles of *Campylobacter* spp. in EU member states. In particular, both *C. jejuni* and *C. coli* from poultry sources in the EU exhibited high resistance against (fluoro-)quinolones and tetracycline, which is in line with our data [6,23,24]. However, notably, the resistance rate to ciprofloxacin and tetracycline was 100% in isolates from industrial poultry samples in Georgia, while in backyard chicken and in human isolates *Campylobacter* strains displayed slightly lower resistance against both antimicrobials. Comparing multi-resistance in *C. jejuni* or *C. coli* in industrial versus backyard chicken, no significant difference could be found. Interestingly, all isolates were sensitive towards gentamicin, chloramphenicol and erythromycin.

Use of (fluoro-)quinolones was shown to be the major risk factor for ciprofloxacin resistance in *Campylobacter* spp. on broiler farms [25]. However, it was shown that the *gyrA* mutation, conferring resistance against (fluoro-)quinolones, can also contribute to a fitness increase in *C. jejuni* in poultry depending on the strain background [26]. The clonal spreading of (fluoro-)quinolone-resistant clones was suggested to occur in Europe [27], although the contribution of whether the resistance was selected through (fluoro-)quinolone use in individual countries and/or transmission between countries is still unclear [28]. Moreover, the differences in resistance rates between the bacterial species from the same source and, therefore, the same antimicrobial exposure indicated that antimicrobial use alone cannot explain differences in resistance profiles of *C. jejuni* and *C. coli* [29]. *C. coli* from the same matrix exhibited higher resistance than *C. jejuni* towards multiple antimicrobials tested [29]. The reason for this phenomenon is still unclear. (Fluoro-)quinolones are among WHOs “Highest Priority Critically Important Antimicrobials” (HPCIA) [30]. Increases in resistance to (fluoro-)quinolones in *Campylobacter* spp. are of concern, as resistance in *Campylobacter* from animals has been shown to be associated with resistance of *Campylobacter* from human infections [6]. When Georgian isolates were compared according to their origin, the chicken *C. coli* or *C. jejuni* isolates were each significantly more resistant towards two and three classes of antimicrobials than the human strains. This might hint to additional infection routes other than cross-contamination from preparing fresh chicken meat and/or direct contact to animals on chicken farms in Georgian children suffering from campylobacteriosis. In addition to the preparation of poultry meat and contact with poultry animals, contact with sand in a sandbox with putative contact to animal feces such as that from dogs and wild animals was also identified in a German study as risk factor positively associated with a *Campylobacter* infection for children under 5 years of age [3].

Furthermore, our study showed a high prevalence of *C.coli* in comparison to *C.jejuni* from poultry samples, which was untypical in a number of countries even in the Caucasus region [6,31,32,33]. However, there are other studies that identified a higher prevalence of *C. coli* than *C. jejuni* in swab samples from farms and neck skins at slaughter in Italy [34] or some alterations of species distribution depending on the stage of broiler production [35]. A long-term study over seven years showed a gradual decrease in the prevalence of *C. jejuni* and a concomittant increase in *C. coli* in cecal samples from chicken in China [36], while in Malaysia both species were frequently isolated from different broiler parts [37].

One explanation for different species distribution might be age and race of the chicken, which is not likely in our study, since we obtained a similar species distribution from backyard chicken of different age and industrial chicken with standardized rearing period of 38–42 days. Our results may additionally hint at the fact that initially, we might have isolated mixed cultures of both *C. jejuni* and *C. coli* in some cases, since PCR results of inoculums identified the presence of both species, which in turn could not be recultivated together.

All tested isolates from Georgia were sensitive towards erythromycin and gentamicin, which was similar for isolates in the EU. Erythromycin resistance in *Campylobacter* isolates from human cases of campylobacteriosis and from broilers in sixteen EU member states was either absent or detected at very low levels in *C. jejuni*, but was observed at higher levels in *C. coli* isolates. Overall, erythromycin resistance was reported in 10% (2020) and 12.9% (2019) of human isolates and 4.4% of broiler isolates. Combined resistance to both ciprofloxacin and erythromycin, which is considered critical for the treatment of campylobacteriosis, was reported to be 8.9% (2020) and 10.4% (2019) in isolates from humans and 4.1% in broilers. In 2020, EU countries reported low prevalence of gentamicin resistance [6]. Data from *C. jejuni* and *C. coli* of human and animal origin in 2019–2020 showed very high to extremely high levels of resistance to (fluoro-)quinolones, which are also critically important antimicrobial agents (CIAs) for the treatment of *Campylobacter* infections in humans [30]. WGS of isolates, especially those with multi-drug resistance, high-level resistance to erythromycin or ciprofloxacin, or resistance to gentamicin or ertapenem, is strongly recommended in order to decipher the antimicrobial resistance determinants involved, their genetic location, and the potential for horizontal transmission [38].

## 5. Conclusions

Preventive and control activities in Georgia are still limited concerning the monitoring and antimicrobial susceptibility profiling of thermotolerant *Campylobacter* spp. Our first national study showed similar AMR patterns of thermotolerant *Campylobacter* spp. strains isolated in Georgia to those reported by the European Union. In particular, resistances against (fluoro-)quinolones and tetracycline were high and should be considered in local therapeutic protocols for severe human cases. Antimicrobial resistance and the prevalence of thermotolerant *Campylobacter* spp. in animals, food and humans need further approaches in order to gain a representative picture of concurrent strains in the Caucasian region.

## Figures and Tables

**Table 1:** Antimicrobial susceptibility of *Campylobacter jejuni* and *Campylobacter coli* strains isolated from three different sources.

Antimicrobial	ECOFF (μg/mL) (R>)		No. (%) of Resistant Isolates							
			Backyard Chicken (*n* = 97)		Industrial Chicken (*n* = 50)		Human (*n* = 57)		Total (*n* = 204)	
	*C. jejuni*	*C. coli*	*C. jejuni* (*n* = 25)	*C. coli* (*n* = 72)	*C. jejuni* (*n* = 5)	*C. coli* (*n* = 45)	*C. jejuni* (*n* = 47)	*C. coli* (*n* = 10)	*C. jejuni* (*n* = 77)	*C. coli* (*n* = 127)
Chloramphenicol	16	16	0	0	0	0	0	0	0	0
Ciprofloxacin	0.5	0.5	20 (80%)	69 (96%)	5 (100%)	45 (100%)	41 (87%)	9 (90%)	66 (86%)	123 (97%)
Erythromycin	4	8	0	0	0	0	0	0	0	0
Ertapenem	0.5	0.5	0	27 (37%)	0	37 (82%)	0	6 (60%)	0	70
Gentamicin	1	1	0	0	0	0	0	0	0	0
Tetracycline	1	2	18 (72%)	52 (72%)	4 (80%)	45 (100%)	17 (36%)	8 (80%)	39 (51%)	105 (83%)

ECOFF, epidemiological cut-off for definition of resistance against antimicrobial substances (EUCAST.org); R>, maximal MIC that represents sensitivity; any MIC exceeding this concentration is defined as resistant. Note that ECOFF for erythromycin and tetracycline differs for *Campylobacter* species. n, number of tested isolates; numbers in table represent numbers of resistant isolates; in brackets, percentage of resistant isolates.

**Table 2:** Association of full susceptibility and resistance to tetracycline and resistance against ≥2 antimicrobials of *Campylobacter* spp. with bacterial species and sample sources.

Anti-Microbial	Covariate	Coefficient of Regression	Standard Error	Wald	Degrees of Freedom	*p*-Value	Odds Ratio	95% Confidence Interval of Odds Ratio		Nagelkerke Pseudo R Squared
								Lower	Upper	
TET	Chicken vs. human	1.153	0.403	8.203	1	0.004	3.167	1.439	6.971	
	*C. coli* vs. *C. jejuni*	0.947	0.391	5.858	1	0.016	2.577	1.197	5.547	0.204
2-3-fold resistance	Chicken vs. human	1.361	0.442	9.487	1	0.002	3.901	1.641	9.276	
	*C. coli* vs. *C. jejuni*	2.271	0.496	21.000	1	<0.001	9.693	3.669	25.607	0.435

TET, tetracycline; Coding of variables: *C. coli* (1) vs. *C. jejuni* (0); poultry isolates (1) vs. human isolates (0).

## Data Availability

Sequence data are available at the Sequence Read Archive (SRA) at the National Centre for Biotechnology Information (NCBI), BioProject No. PRJNA844526, Bio Sample Accession No. SAMN28822301- SAMN28822340. Further data that support the findings of this study are available on request from the corresponding authors.

## Supplementary Materials

The following supporting information can be downloaded at: https://www.mdpi.com/article/10.3390/antibiotics11101419/s1, Table S1: Complete sample list, including phenotype of antibiotic resistance; Table S2: WGS data overview and antibiotic resistance of tested samples; Table S3: WGS data-plasmid annotation.
